# Gastrointestinal Strongyles Egg Excretion in Relation to Age, Gender, and Management of Horses in Italy

**DOI:** 10.3390/ani10122283

**Published:** 2020-12-03

**Authors:** Antonio Scala, Claudia Tamponi, Giuliana Sanna, Giulio Predieri, Giorgia Dessì, Giampietro Sedda, Francesco Buono, Maria Grazia Cappai, Vincenzo Veneziano, Antonio Varcasia

**Affiliations:** 1 Department of Veterinary Medicine, University of Sassari, 07100 Sassari, Italy; scala@uniss.it (A.S.); claudiatamponi@yahoo.it (C.T.); giulianasanna@yahoo.it (G.S.); giorgia.dessi87@tiscali.it (G.D.); giampisedda@gmail.com (G.S.); mgcappai@uniss.it (M.G.C.); 2 ACME S.r.l., Corte Tegge, 42025 Cavriago RE, Italy; giulio.predieri@acmedrugs.com; 3 Department of Veterinary Medicine and Animal Productions, University of Naples Federico II, 80138 Naples, Italy; francesco.buono@unina.it (F.B.); vinvene@unina.it (V.V.)

**Keywords:** gastrointestinal strongyles, prevalence, intestinal parasites, horses, fecal egg shedding, management, seasonality

## Abstract

**Simple Summary:**

Horses worldwide are plagued by gastrointestinal parasites that can lead to severe health problems. The occurrence and intensity of these worm infections vary based on the geographical location, season, and animal management strategies applied. One of these strategies is to monitor the parasite situation in different parts of the world. This research investigates the abundance, proportions, and risk factors of strongyle egg shedding of horses in Italy. Overall, the results showed that approximately 40% of all horses in Italy shed strongyle eggs and that almost 90% of stables have at least one infected animal. In addition, most parasite eggs are found in just a small fraction of the horse population, confirming the need for improved parasite control strategies.

**Abstract:**

Current equine helminth control strategies play a key role in strongyle epidemiology and anthelmintic resistance and have led to the recommendation for new treatment plans, which include diagnostic and efficacy surveillance. Assessing the equine strongyle distribution patterns would thus be useful and this study describes the strongyle prevalence in the equine population in Italy through coprological analysis and coproculture. In addition, individual data on each animal were collected in order to identify risk factors associated with strongyle egg shedding. Of the total number of stables investigated, 86.4% were found to have at least one positive animal and a 39.5% prevalence of strongyle egg shedding with a mean eggs per gram (EPG) of 245. A total of 80% of the total recorded EPG was shed from 12.8% of positive horses, thus confirming the need for new targeted intervention strategies. Significant differences in parasite prevalence were found based on season, sex, geographical distribution, management and rearing system, and breed. Significantly lower EPG values were found in horses that had received anthelmintic treatment, and macrocyclic lactones (MLs) were the most effective. Lastly, although large strongyles are more pathogenic, horses in Italy are mainly burdened by small strongyles, which pose an important animal health risk requiring continuous parasitological monitoring.

## 1. Introduction

Equids are exposed to a large number of helminths throughout their lives [1], above all gastrointestinal strongyles (GIS) (large and small strongyles). GIS infections are an important concern considering their worldwide distribution and negative impact on animal health and performance [2,3].

Although horses can harbor significant worm burdens without showing clinical symptoms, specific syndromes have been ascribed to infections by cyathostomins and large strongyles [4,5].

Because of their high pathogenic role, large strongyles, especially *Strongylus vulgaris*, were used as the primary target of anthelmintic treatment. Although, today, they are no longer commonly reported in domestic horses [6,7], selective therapy has been associated with an increased prevalence of this parasite [6].

Cyathostomins (small strongyles), which are less pathogenic, but more common and abundant, are considered the most problematic group of helminths [4,8], as they were blamed for about 10% of deaths in animals [9]. The mass emergence of cyathostomin larvae in the large intestines can cause larval cyathostominosis, which is characterized by watery diarrhea, dehydration, and ventral oedema, with a case-fatality rate of around 50% [4,7,10]. Because of their large diffusion, cyathostomin parasites are considered the main targets of parasite control strategies [6,7].

Parasite control practices used to be based on frequent treatments at fixed times (interval dosing programs and strategic dosing programs) without diagnostic surveillance [11]. Today, the general recommendation proposed by the Parasite Control Guidelines of the American Association of Equine Practitioners (AAEP) is to use a strongyle fecal egg count (FEC) to estimate the levels of egg shedding by each horse and to evaluate anthelmintic treatment efficacy using the fecal egg count reduction test (FECRT) [12].

Strongylosis is also controlled through good management practices and environmental prophylaxis measures such as the removal of feces from boxes and paddocks, and pasture rotation [13], which can decrease the risk of dangers associated with equine management conditions for a number of reasons often associated with the latitude and season as well as the animal response and environmental adaptation [14,15,16].

However, anthelmintic treatments are the most widespread and effective strategies to control helminth infections in horses [13,17,18].

The increasing levels of anthelmintic resistance in equine cyathostomins worldwide make the design of adequate control programs very challenging [19]. Of the three modern anthelmintic drug classes licensed for use in equines [20], benzimidazoles (BZs), tetrahydropyrimidines (THPs), and macrocyclic lactones (MLs), widespread cyathostomin resistance has been recorded for BZs and THPs, with emerging resistance to MLs reported as a shortened egg-reappearance period (ERP) in horses and in donkeys [19,21]. Some reports suggest that a shortened ERP is caused by resistant luminal L4 stages, which survive treatment and mature into egg-laying adults [22,23,24].

Assessing the helminth distribution patterns in managed equine populations would provide useful information for developing tailored control methods that are less reliant on chemical compounds [8], such as the use of medicinal plants, although there are few data that prove their effectiveness [25].

All equids should be screened regularly, however, only those exceeding a predetermined strongyle FEC threshold (200 eggs per gram of feces (EPG)) are treated (selective treatment) [26,27,28]. In fact, strongyle egg counts are typically over-dispersed among horses [8,29], and mature horses are likely to consistently maintain their typical egg shedding level over time [30,31,32].

This means that a relatively small proportion of horses within a group or in a stud are responsible for spreading the majority of the total strongyle egg output, and that the same horses are the primary contributors to pasture contamination. This has also been described as the ‘20/80 rule’, with approximately 20% of horses responsible for 80% of the total strongyle egg output [29,33,34]. Thus, it is imperative to identify these “high egg shedders” and ensure that they get treated in order to reduce the infection pressure [8,33,35].

A few recent surveys on horses in some regions of Italy reported a decrease in strongyle infection from 67.4% [36] to 47.6% [37]. Apart from these two papers, no other large-scale and comprehensive surveys on gastrointestinal strongyles in Italy have been carried out.

The aims of this study were thus to describe strongyle parasite egg shedding patterns in the equine population in Italy, in order to update the epidemiological scenario, as well as to identify risk factors associated with gastrointestinal strongyle infection.

## 2. Materials and Methods

### 2.1. Study Animals

A total of 6896 horses, with age ranging from 6 months to 44 years (mean age 9.2 ± 6.5 years), from 548 different stables located all throughout Italy were examined for the presence of strongyle egg shedding between January 2015 and December 2016.

Forms were completed for individual samples with information on the following:

(a) age, sex, breed, and stable;
(b) environment, period of the season when sampling occurred, and geographical area of the stable;
(c) management: individual stable only and/or with outdoor access or exclusively outdoors without night shelter; equestrian discipline; and, finally, treatments against parasites performed in the previous six months prior to the sampling time.

Data were entered on a spreadsheet (Microsoft Excel Vers. 16.16.3) for statistical analysis.

### 2.2. Coprological Analysis

In accordance with Nielsen et al. [5], individual fecal samples were collected directly from the rectum of each horse during routine veterinary practice and sent to the laboratory within a maximum of three days from collection. They were vacuum-packed and delivered by express courier in refrigerated boxes (about 4 °C).

Individual FEC was carried out using McMaster slides according to Raynaud [38] using a sodium chloride (NaCl) supersaturated solution (specific gravity 1.2) as a flotation fluid, with a detection limit of 15 EPG.

On the basis of the laboratory analysis outcome, horses were allocated into five classes according to the egg excretion, as suggested by Ambrosi [39] and Euzeby [40]:

(1) <200 EPG, low infestation;
(2) between >200 and 500 EPG, mild infestation;
(3) between >500 and 600 EPG, moderate infestation;
(4) between >600 and 1000 EPG high infestation;
(5) >1000 EPG, severe infestation.

### 2.3. Coprocultures

Pooled coprocultures were performed using fecal samples from positive animals, including horses with FEC > 1000 EPG and incubated at 25–27 °C for 10 days in order to obtain third stage larvae (L3). These were then isolated with the Baermann technique [41]. They were then identified according to their specific genera and/or species, using the morphological keys proposed by the Atlas of Diagnosis of Equine Strongylidosis [42].

### 2.4. Statistical Analysis

All data were processed for statistical analysis. For ease of interpretation, the age of animals was converted into a class of age according to Relf et al. [11] adapted as follows: younger than 1 year (foal), age class = 1; 2 to 4 years, age class = 2; 5 to 14, age class = 3; 15 to 21, age class = 4; and older than 21, age class = 5.

Similarly, data on the origin of samples were grouped into three geographical macro-areas: northern Italy (Piedmont, Lombardy, Trentino Alto Adige, Friuli Venezia Giulia, Veneto, Emilia Romagna, Liguria, and Valle d’Aosta); central Italy (Tuscany, Umbria, Marche, Lazio, Abruzzo, and Molise); and southern Italy and the islands (Campania, Basilicata, Puglia, Calabria, Sicily, and Sardinia).

Non-parametric variables were processed with the Mann–Whitney test and Kruskal–Wallis test.

When continuous variables were obtained in subsets of data, EPG classes were analysed according to the following general model:

Yi, j,k = μ + Di,j + Gj, k + Fi,k + Di,j × Gj,k + ei, j, k(1)

where Y is the dependent variable (EPG class, five levels: low, mild, moderate, high, and severe infestation); μ is the overall mean; D is the fixed effect of the sampling time (season, four levels: spring, summer, fall, and winter); G is the fixed effect of the region (three levels: northern, central, and southern); F is the fixed effect of the stud; D × G is the interaction factor; and e is the random residual.

Samples with incomplete information were analyzed by one-way analysis of variance (ANOVA) for gender, anthelmintic treatment, and management. Confidence intervals and groupings were adjusted according to the Tukey method. Statistical significance was set at *p*-value < 0.05 using Minitab^®^, (Minitab 18, Inc., State College, PA, USA) and Epi-Info^®^ 6.0 (CDC/WHO, Atlanta, GA, USA).

### 2.5. Ethical Statement

The study was carried out following the recommendations of the European Council Directive (86/609/EEC) on the protection of animals. Ethical approval was not required in this study, as fecal sampling was performed by the vet as part of the routine clinical visit.

## 3. Results

An overall prevalence of 39.5% (2727/6896; 95% confidence interval (CI): 38.3–40.7) of GIS eggs was found in the animals examined. An EPG mean ± standard deviation (SD) of 245 ± 661 and a mean intensity (MI) ± SD of 620 ± 933.2 were recorded.

Among the examined stables, 86.4% (95% CI: 83.6–89.4) showed at least one positive horse for GIS, and in 68.4% of the stables (95% CI: 64.5–72.3), animals with FEC > 200 EPG were recorded.

Table 1 shows the prevalence values of horses grouped into EPG classes, which also shows that 21.4% of the horses examined had an FEC > 200 EPG.

Data processed by year showed prevalence rates of 42.1% in 2015 (95% CI: 40.9–43.3) and 35.9% in 2016 (95% CI: 34.8–37.0) (χ2 = 27.23; *p* = 0.0000013), with EPG means of 259.2 ± 664.3 and 225.9 ± 654.8, respectively (Mann–Whitney test, W = 14,210,577; *p* = 0.0001), and mean intensity (MI) of 614.9 ± 910.2 and 629.9 ± 970.0, respectively (Mann–Whitney test, W = 2,306,920; *p* = 0.906).

Regarding the “80/20 rule”, the present study shows that the 80% of all EPG values recorded (1,691,397) were shed from 12.8% of positive horses, the highest shedders, in which the EPG ranged from 645 to 10,770.

The seasonal prevalence rates differed significantly (χ2 = 22.244, *p* < 0.00001), highlighting a greater number of positive animals in winter (43.1%), with a 1.48 times higher probability of horses being positive for GIS compared with the summer, when the prevalence rates were lower (33.8%). A significant effect of sampling season was observed on EPG levels (*p* < 0.0001). Higher EPG values were found in the winter and in spring (*p* < 0.0001), when the highest mean intensity (MI) values were also recorded. The results stratified by sampling season are reported in Table 2.

Unfortunately, not all samples were accompanied by complete animal data (information on sex, management, breed, last deworming), thus the statistical analysis of these variables was performed on a subset of data.

The sex effect on EPG (in a subset of 5137 animals: 2366 females and 2771 males) highlighted significantly higher rates in females compared with males (χ^2^ = 10.66; *p* = 0.0001). Detailed results are reported in Table 2.

A significant difference was also found between the GIS prevalence rates for animals from the three different geographical areas examined (χ^2^ = 51.1, *p* < 0.0001) (Table 2).

The non-parametric Kruskal–Wallis test highlighted significant differences in EPGs from animals in the three areas of Italy considered, and highlighted significantly higher EPG levels in central Italy (H = 53.05; *p* = 0.0001).

Data stratification relative to 5696 horses for which information was available regarding management (indoor, outdoor, in/outdoor) highlighted significant differences between the GIS prevalence (χ^2^ = 74.16, *p* < 0.0001). The highest prevalence was found in horses reared outdoors (49.7%) (Table 2).

The EPG means recorded in horses reared with different methods were also significant (*p* < 0.0001). The highest EPG means were found in horses reared outdoors (369.7 EPG).

Data processed by age groups obtained from 5077 horses are shown in Table 3. Prevalence rates decreased significantly in older age groups (χ^2^ for linear trend 145,203; *p* < 0.0001), as well as odds ratio values. EPG levels correlated negatively with age (Pearson’s R = −0.110; *p* < 0.001), and the highest EPG values were recorded in horses aged between 1 and 4 years (*p* < 0.0001).

The analysis of data according to breed included a total of 4423 horses (Table 4). EPG prevalence rates highlighted significant differences according to breed (*p* < 0.001), with rates observed in descending order for Italian autochthonous (63%), South American (55.5%), and trotters (49%).

Coprocultures carried out on 68 positive farms enabled a total of 7385 larvae L3 to be isolated and classified, showing the presence of eight different genus/species: *Cyatostomum* spp. (79.4%), *Strongylus vulgaris* (10.3%), *Trichostrongylus axei* (7.1%), *Oesophagodontus* sp. (1.0%), *Triodonthophorus* spp. (0.9%), *Strongylus equinus* (0.6%), *Gyalocephalus capitatus* (0.5%), and *Strongylus edentatus* (0.2%). Details on the identification of L3 larvae are shown in Table 5.

Of the 6896 horses examined, 4468 had received an anthelmintic treatment in the previous six months (Table 6), and of these, 64.5% of the animals were treated with MLs, followed by THPs (25.7%) and BZs (7.2%). Only 0.2% of treatments were performed using a herbal dewormer (Elmene LX^®^, Agrolabo, Scarmagno, Italy) containing *Trachyspermum ammi*, *Mallotus philippinensis*, *Cassia angustifolia*, *Chenopodium* spp., *Azadirachta indica*, *Butea monosperma*, and *Artemisia absinthium*).

Significantly higher EPG levels were reported in animals that had not been dewormed in the previous six months (*p* < 0.0001). In addition, there was a significant effect of the anthelmintic class on the average EPG value (*p* < 0.0001). The lowest FEC values were found in horses that had been previously treated with MLs.

## 4. Discussion

We collected data on equine gastrointestinal strongyle egg shedding for one specific date in a national horse population in Italy.

The results show that GIS prevalence rates (39.5%) detected in our survey are currently lower than those reported in other recent surveys carried out in Italy, which found a cyathostomin prevalence of 67.4% [36] and 47.6% [37]. Concerning horses shedding more than 200 EPG (21.4%), our findings are in the middle, between 12% found by Sconza et al. [37] and 42.2% reported by Traversa et al. [36].

The moderate egg shedding value that we identified could be associated with two main factors: (i) the time between the last anthelmintic treatment and fecal egg count; (ii) encysted and arrested larvae of small strongyles that can be responsible for a negative FEC, which then becomes positive over time also without reinfestation.

It should also be highlighted that a low FEC does not always correspond to a low infection level. No linear correlation between FECs and the parasite burden has been found, as high levels of egg shedding are not necessarily equivalent to an increased risk of clinical disease [28].

Our survey shows that, in 68.4% of the stables, horses had an FEC > 200 EPG, which is the suggested anthelmintic treatment cut-off [29,40]. These EPG levels were found in 21.4% of the analysed animals, confirming that just a small percentage of horses are responsible for pasture contamination [43]. In fact, we found that only 12.8% of the sampled horses were responsible for excreting 80% of the strongyle eggs, in line with the generally accepted 80/20 rule [8,33]. This is important because this pattern is at the foundation of current recommendations regarding equine parasite control, where the egg shedding level determines how intensively a given equid should be exposed to an anthelmintic drug [13], and only horses that exceed an FEC value of 200 EPG should be treated (selective treatment).

Cyathostomins are the most common GIS parasites in Italy [36,37], with a prevalence in the monitored stables of 98.5%. However, the presence of *S. vulgaris* in 79.4% of the stables merits particular attention because of its important pathogenic role in horses. A survey carried out on horses in Sardinia showed lesions to the mesenteric arteries in 100% of the animals examined, despite being detected in only 41% of the fecal samples from the same animals [44]. Moreover, non-strangulating intestinal infarction may be associated with the migration of the larval stage of *S. vulgaris* [45]. It is thus important to ascertain the presence of large strongyles, especially *S. vulgaris*, which also entails an anthelmintic treatment with a macrocyclic lactone in low contaminators (FEC < 200 EPG) in order to prevent the spread of this large strongyle.

The presence of *S. vulgaris* in the stables screened in this survey suggests that anthelmintic treatments should be carried out once or twice a year [6], because of the prepatent period of this nematode [39].

We found that the most important significant associations for the onset of GIS in horses were the following: season, sex, age, and type of breeding.

The season seems to significantly influence the trend of GIS, in fact, there was an increase in prevalence rates during colder periods, while the highest EPG mean and MI values were recorded in the spring. During the summer, in contrast with the literature [46,47], EPG values were significantly lower. Further studies are needed to better investigate this unusual finding. One model regarding the development of third stage larvae of small strongyles reported that, under cold temperatures, eggs did not develop into L3, but were able to survive for a long period and resume development when weather conditions were better [47]. On the other hand, under tropical conditions, the development of eggs is rapid and a large number of L3 are produced, but do not survive for long [47]. Kornas et al. [46] showed an influence, albeit not very high, of the climate on EPG values. High temperature values may lead to an increase, while abundant rainfall seems to decrease the EPG values.

Other studies carried out in northeast Italy revealed no significant differences between prevalence rates found in horses examined in the summer and in winter [48].

In this study, the time since the last deworming and coprological examination affected the equine strongyle egg count, as also reported by Kornas et al. [46] and Scare et al. [49]. In addition, horses treated with macrocyclic lactones showed a lower FEC than those treated with other anthelmintic drug classes, as also reported by Nielsen et al. [35].

Data stratified by sex showed a significantly higher prevalence in females as reported in other surveys [13,31], indicating it is likely that pregnancy/birth and lactation cause stress, which makes females more sensitive than males. This may also be determined by the close and longer contact between the mares and their foals, in fact, foals are more efficient at shedding eggs [46,50]. This could also be explained by the fact that stallions have less access to pasture and are thus at minor risk of infection.

Our results show that younger equids (≤4 years) had a higher FEC than adults, in accordance with Hoglund et al. [51] in Sweden, who showed that 1–5-year-old horses shed significantly more GIS eggs than older horses. Similar results have also been reported in the United States, Denmark, and Spain [13,52,53]. This suggests, therefore, that young animals could be considered as markers of these infections and should thus be carefully monitored.

We also found significant variations in the prevalence rates in horses of different breeds, in agreement with Kornas et al. [46]. However, a plausible explanation could be related to the different management of the animals, rather than to a potential genetic susceptibility.

Across the country, there were also significant differences regarding the GIS prevalence rates recorded in the north, center, and south (plus islands), as well as significantly higher EPGs found in central Italy, where the highest prevalence (46.3%) and odds ratio (1.44) were recorded.

The type of breeding also influences the parasite trend. We found that animals reared outdoors or animals confined in paddocks represent an important risk factor for GIS, as also reported by Kornas et al. [46]. In such breeding conditions, the periodical removal of feces (ideally twice a week) could thus be a particularly useful prophylactic measure, thus reducing the contamination of intestinal strongyle larvae [54].

Concerning the use of anthelmintics in horses included in this survey, our findings showed that, to date, ivermectin is the most common drug used to control GIS in horses in Italy, which is in line with other studies [35,55].

Anthelmintic treatment should be performed only after diagnosis and targeting horses with an FEC > 200 EPG (selective therapy), as well as performing the FECRT 14 days post treatment to evaluate the anthelmintic efficacy [35,56].

The active principles should be chosen carefully, considering at which parasitic species and which state of infection the anthelmintic treatment is aimed. Rotating dewormers do not slow the development of anthelmintic resistance, as they do not prevent the accumulation of resistant genes [57].

## 5. Conclusions

Our study has shown that gastrointestinal strongyles, above all small strongyles, are still widespread in Italy, with prevalence rates of 39.5% among the horses monitored, and with 21.4% of horses shedding more than 200 EPGs.

The statistical analysis identified several risk factors, including sex, age, season, and type of breeding. A higher prevalence was thus found in females, in horses aged under five years, in the winter season, and in central Italy.

These findings highlight the need to improve control strategies for gastrointestinal strongyles in Italy, by applying targeted treatments recommended by experts in the field. Regular FEC monitoring should be performed in order to identify horses with egg levels above 200, which should receive anthelmintic treatments. Particular attention should be paid to the selection of drug treatment, in order to make more rational use of the anthelmintic classes, avoiding the use of the same molecule for repeated treatments, and favoring those drugs for which anthelmintic resistance has not been reported in the considered area of Italy.

Finally, parasite control strategies should become less dependent on anthelmintic use and more reliant on management-based control within equine rearing practices.

## Figures and Tables

**Table 1:** Prevalence rates reported by Eggs Per Gram (EPG) classes.

EPG Classes	EPG Values	Horses	Prevalence
0	0	4169	60.5%
1	<200 EPG	1249	18.1%
2	>200 ≤ 500 EPG	514	7.4%
3	>500 ≤ 600 EPG	115	1.7%
4	>600 ≤ 1000 EPG	299	4.3%
5	>1000 EPG	550	8.0%

**Table 2:** Prevalence rates, mean value and mean intensity (MI) of Gastro Intestinal Strongyles (GIS) EPG found in examined horses and their odds ratios (ORs) reported for different seasons, sex, zone and type of breeding.

Elaboration	Seasons				Sex (Data Available for 5137 Horses)		Zone			Type of Breeding (Data Available for 5696 Horses)		
	Summer	Autumn	Winter	Spring	Male	Female	Northern Italy	Central Italy	Southern Italy and Islands	Indoor	Outdoor	In/ Outdoor
Examined	1230	1646	1937	2083	2771	2366	4118	1878	900	2,383	949	2364
Prevalence %	33.8	37.6	43.1	41.2	35.8	40.2	37.4	46.3	35.2	33.7	49.7	39.2
EPG Mean	211.5	217.8	250.1	282.4	207.41	258.11	226.7	284.1	249.1	172.1	369.7	248.9
MI * EPG	625.2	579.2	580.9	685.7	580	642.16	606.3	383.3	712.0	510.9	743.4	634.8
Odds Ratio (OR)	1.00	1.18	1.48	1.37	1.00	1.05	1.00	1.44	0.91	1.00	1.95	1.27

* MI = mean intensity.

**Table 3:** Prevalence rates for GIS and mean EPG reported for different age groups and their statistical analysis, on a subset of 5077 horses.

Age Groups	Examined	Prevalence %	Odds Ratio	EPG Mean	Standard Deviation
<1 year	128	49.2	1.00	345.4	803.3
1 year	365	55.1	1.26	632.2	1156.0
2–4 years	985	53.1	1.17	383.3	774.9
5–14 years	2506	34.8	0.55	140.8	411.4
15–21 years	847	27.5	0.39	143.2	435.2
>22 years	246	32.1	0.49	209.5	611.7

Note: Chi Square for linear trend = 145.203; *p* < 0.0001.

**Table 4:** Statistical analysis of prevalence rates and ranges of EPG reported for horses of different breeds and/or groups of breed.

Breed	Examined Horses	Prevalence %	≤500 EPG %	>500 EPG %	Odds Ratio
English Thoroughbred	332	45.2	29.5	15.7	1.00
Arabian Thoroughbred	212	37.3	26.9	10.4	0.524
Half-breed horses	34	44.1	23.5	20.6	0.958
Trotter	906	49.0	29.9	19.1	1.166
American Breed	555	45.8	28.3	17.5	1.024
South-American Breed	18	55.5	27.8	27.8	1.517
Spanish Breed	31	16.1	12.9	3.2	0.233
Italian Breed	84	63.0	39.3	23.8	2.074
Jumping	2014	31.3	21.4	9.9	0.554
Ponies	237	47.3	32.1	15.2	1.087

**Table 5:** Prevalence of different larval species (L3) of intestinal strongyles found in 68 positive stables.

Parasites Species	% Recovered Larvae	% Positives Stables
*Cyathostomum* spp.	79.4	98.5
*Strongylus vulgaris*	10.3	79.4
*Trichostrongylus axei*	7.1	61.8
*Oesophagodontus* spp.	1.0	33.8
*Triodonthophorus* spp.	0.9	13.2
*Gyalocephalus* spp.	0.5	26.5
*Strongylus equinus*	0.6	16.2
*Strongylus edentatus*	0.2	4.4

**Table 6:** Drugs administered in 4468 of examined horses with a history of deworming in the previous six months.

Drugs	Number of Treatment	Prevalence	EPG Mean
Macrocyclic Lactones (MLs)	2882	32.8%	188.4 ± 536.8
Benzimidazoles (BZs)	323	24.1%	372.5 ± 910.1
Tetrahydropyrimidines (THPs)	1150	43.8%	262.3 ± 612.6
Elmene LX *	9	44.4%	371.7 ± 661.0
Unknown	104	47.1%	544.0 ± 867.8

* = commercial product formulated as a liquid nutritive integrator containing a mix of vegetal (*Trachyspermum ammi, Mallotus philippinensis, Cassia angustifolia, Chenopodium spp., Azadirachta indica, Butea monosperma, Artemisia absinthium*).
